# Cell Fate Analysis of Embryonic Ventral Mesencephalic Grafts in the 6-OHDA Model of Parkinson's Disease

**DOI:** 10.1371/journal.pone.0050178

**Published:** 2012-11-29

**Authors:** Sonya Carvalho Neto, Ahmad Salti, Zoe Puschban, Nadia Stefanova, Roxana Nat, Georg Dechant, Gregor K. Wenning

**Affiliations:** 1 Division of Neurobiology, Department of Neurology, Medical University Innsbruck, Innsbruck, Austria; 2 Institute for Neuroscience, Medical University Innsbruck, Innsbruck, Austria; “Mario Negri” Institute for Pharmacological Research, Italy

## Abstract

Evidence from carefully conducted open label clinical trials suggested that therapeutic benefit can be achieved by grafting fetal dopaminergic (DAergic) neurons derived from ventral mesencephalon (VM) into the denervated striatum of Parkinson's disease (PD) patients. However, two double-blind trials generated negative results reporting deleterious side effects such as prominent dyskinesias. Heterogeneous composition of VM grafts is likely to account for suboptimal clinical efficacy.

We consider that gene expression patterns of the VM tissue needs to be better understood by comparing the genetic signature of the surviving and functioning grafts with the cell suspensions used for transplantation. In addition, it is crucial to assess whether the grafted cells exhibit the DAergic phenotype of adult substantia nigra pars compacta (SNpc). To investigate this further, we used a GFP reporter mouse as source of VM tissue that enabled the detection and dissection of the grafts 6 weeks post implantation. A comparative gene expression analysis of the VM cell suspension and grafts revealed that VM grafts continue to differentiate post-implantation. In addition, implanted grafts showed a mature SNpc-like molecular DAergic phenotype with similar expression levels of TH, Vmat2 and Dat. However, by comparing gene expression of the adult SNpc with dissected grafts we detected a higher expression of progenitor markers in the grafts. Finally, when compared to the VM cell suspension, post-grafting there was a higher expression of markers inherent to glia and other neuronal populations.

In summary, our data highlight the dynamic development of distinctive DAergic and non-DAergic gene expression markers associated with the maturation of VM grafts in vivo. The molecular signature of VM grafts and its functional relevance should be further explored in future studies aimed at the optimization of DAergic cell therapy approaches in PD.

## Introduction

Parkinson's disease (PD) is a neurodegenerative disorder characterized by a progressive loss of dopaminergic (DAergic) neurons of the substantia nigra pars compacta (SNpc) with a subsequent reduction in striatal dopamine content accounting for parkinsonian features such as bradykinesia, rigidity and tremor at rest [1]. Levodopa (L-DOPA) continues to be the most effective pharmacologic treatment for the alleviation of motor symptoms [2], whereas cell replacement therapies aim at brain repair, restoring nigrostriatal DAergic transmission [3]. Studies in animal models of PD provide the proof of principle of DAergic cell therapy showing that grafted DAergic neurons reinnervate the denervated striatum and make synaptic contact, release dopamine, receive inputs from host neurons and improve motor deficits [4]. These experiments led to a series of open label clinical trials demonstrating meaningful and sustained clinical benefit derived from DAergic rich fetal ventral mesencephalic (VM) grafts [5]. However, in two double-blind randomized sham-controlled trials, several patients developed graft-induced dyskinesias (GID) and this has seriously limited further development of DAergic cell replacement therapies for PD [6], [7].

It has been proposed that GID could be associated to a failure of the grafts to restore DAergic synaptic contacts with the host striatal neurons [8] or to an inflammatory and immune responses around the graft, since side effects develop either in cases where there was no immunosupression [6] or after discontinuation of the treatment [9], [10]. However, serotonergic (5-HT) neurons, that in developmental stages appear caudally to the VM and could be cografted, have also been implicated in the development of GID [11]. Studies in animal models demonstrated that GID are not necessary related to the absolute number of 5-HT neurons cografted but instead to the ratio of serotonin/dopamine innervation of the host striatum [12]. In patients transplanted with fetal mesencephalon that developed GID after some years of exhibiting major motor recovery, GID were correlated with a hyperinnervation of the host striatum by 5-HT neurons [13].

Besides, understanding what is the exact phenotype within the midbrain dopaminergic (mDAergic) neuronal population of the VM grafts that gives rise to the functional DAergic neurons post transplantation has been a matter of discussion [14]. Sinclar et al, 1999 [15] showed that proliferating DAergic progenitor cells were not able to differentiate *in vivo*, however, others [16]–[18] demonstrated that VM tissue dissected at an embryonic age where the majority of the cells is expected to still undergo cell division were able to finalize the differentiation process post-implantation. In addition, it was shown that survival rates of DAergic neurons could be increased 5-fold just by transplanting VM tissue from younger embryos [16].

mDAergic neurons develop at the ventral midbrain floor plate. Many of key players responsible for genetic cascades involved in the induction of mDAergic neuron progenitors, specification of mDAergic precursors and maintenance of postmitotic mDAergic neurons have been identified [19]–[24]. Early in development (E8–E9 in mice), specification of mDAergic progenitor is induced by extracellular gradients of fibroblast growth factor 8 (Fgf8) from the midbrain-hindbrain boundary and sonic hedgehog (Shh) from the notochord [25]. These factors act together with WNT-1, -3a, and -5a for the induction/repression of many of the transcriptional factors responsible for mDAergic specification and differentiation [26]. Proliferating mDAergic progenitors are characterized by combined expression of Otx2, Lmx1a/b, En1/2, Msx1/2, Ngn2 and Mash1 [23]. As progenitors become postmitotic, they start to express Nurr1, a transcription factor that controls DAergic neuron transmitter phenotype and subsequently Pitx3, the most specific mDAergic neuronal marker [27]. Final differentiation is characterized by the expression of TH and other markers related to synthesis, storage, release and reuptake of dopamine such as AaDc, Vmat2, D2 and Dat [22].

Although not optimal, VM grafts continue to be regarded the “gold standard” for transplantation in PD [28]. We hypothesized that a greater knowledge of gene expression profiles of VM grafts will generate important insights into VM graft development, composition, and functional benefit. In addition, it is essential to establish whether VM grafts resemble DAergic neurons of SNpc, so that dopamine replacement is performed in the most physiological way. To this end, we grafted embryonic VM cell suspensions from a green fluorescent protein (GFP) reporter mouse into a 6-hydroxydopamine (6-OHDA) rat model. GFP expressing VM grafts were dissected from the host striatum and a comparative analysis of the molecular signature of the graft tissue, before and after transplantation, was conducted using qRT-PCR. In addition, VM grafts were compared to fully differentiated SNpc DAergic neurons.

## Methods

### Experimental design

Adult (200–250 g) Wistar rats served as graft recipients in this study. All animal experiments were performed according to the Austrian guidelines for the care and use of laboratory animals and all experiments were approved by the Federal Ministry for Education, Science and Research of Austria with the reference number do.ZI. 6545. Animals were housed on a 12/12 h light/dark cycle with free access to food and water. All efforts were made to minimize the number of animals used and their suffering. Euthanasia was assessed as the first signs of pain or distress were manifested.

The animals were subjected to a unilateral 6-OHDA lesion and two weeks after lesions, amphetamine induced rotation was inspected. Only animals with post-lesion net ipsilateral scores of 6 turns/min per session were selected for transplantation surgery. These animals were divided into two groups: sham-grafted (n = 7) and transplantation (n = 20). Transplantation group received VM embryonic tissue (12.5-day old embryos) derived from time mated mice expressing enhanced GFP. The sham group on the other hand received medium in which VM cell suspension was transplanted. In the 6^th^ week post-transplantation, animals displaying more than 40% reduction (n = 15) of amphetamine-induced rotations and sham-grafted animals were randomly subdivided into two sub groups: the immunohistochemistry (IHC) group (n = 8) and the qRT-PCR group (n = 7). In the IHC group the animals were perfused transcardially, the brains were serially cut and processed for tyrosine hydroxylase (TH), GFP and glial fibrillary acidic protein (GFAP) staining. In the qRT-PCR group the brains were cut fresh into slices and grafts were dissected with the use of a high magnification fluorescent microscope (Leica). The grafts were catapulted into lysis buffer for RNA extraction and qRT-PCR quantification was performed.

In order to have gene expression characterization of the grafts prior to transplantation a sample was taken from the remaining suspension of cells used for transplantation procedure and further characterized by qRT-PCR. A second sample was taken and the cells were smeared on microscope slides in order to count TH cells pre-grafting.

### Animal model of Parkinson's disease

Under isoflurane anaesthesia (induction 3.5%, follow-up 1.5–2%), the animals received a unilateral injection of 6-OHDA (2 µg/µl in 0.9% saline with 0.1% ascorbic acid; Sigma) into the left medial forebrain bundle using a stereotaxic apparatus and a 10 µl Hamilton syringe. The stereotactic coordinates used were: AP −2.2; L +1.5; V −8.0 (in mm from bregma and dura) with the tooth bar set at +4.5 [29]. A total of 4 µl of the toxin was injected at a rate of 1 µl/min and the needle was kept in the brain for additional 2 minutes to allow a complete diffusion of the toxin before it was slowly lifted.

### Amphetamine-induced rotation

Rotation testing under the influence of amphetamine (Sigma) was assessed two weeks after lesion using 2.5 mg/kg injected intra peritoneally (i.p.). Test scores were accumulated over 90 min test sessions in order to estimate the extent of striatal dopamine denervation. The collected data are reported as net turns per minute (ipsilateral minus contralateral) over the full session. Only rats with post-lesion net turns of ≥6 turns/min/session underwent transplantation surgery. The remaining animals were euthanized with the use of a mixture of 75% CO_2_ and 25% of O_2_. Grafted and sham-grafted animals were retested 6 weeks post-implantation using the same method.

### VM cell suspension and transplantation surgery

Time pregnant mice expressing GFP under the direction of the chicken beta-actin promoter (C57BL/6(CAG-EGFP) C14-Y01-FM131Osb/RIKEN Bio Resource Centre, Ibaraki, Japan) were used as a source of embryonic VM tissue for transplantation. The morning after overnight pairing was regarded as day 0.5 of gestation. Pregnant females were killed by cervical dislocation on day 12.5 of gestation and embryos were removed. Donor ages were confirmed with crown-rump length (CRL) measurements. Only embryos with CRL of 8.5 mm+/−0.5 were used as donor tissue for transplantation. Tissue was processed using standard protocols [30]. Briefly, pieces were collected in 0.6% glucose-saline, incubated with 0.1% trypsin and 0,05% DNase, tissue pieces were washed and mechanically dissociated using fire polished Pasteur-pipettes with decreasing inner tip diameters. Viability of cells was determined using trypan blue dye exclusion. Cells were resuspended in a DNase solution (0.05% DNase in DMEM/F12, Gibco) at a density of 200.000 cells/µl and kept on ice until use. The transplantation procedure was performed according to standard transplantation protocol [31]. A total of 2 µl of cell suspension was injected into the striatum in 2 deposits at the following coordinates: AP 0.48; L 2.2; V1 −5.5; V2 −4.5; tooth bar −3.3. From grafting until the end of the study all animals received daily i.p. injections of cyclosporine-A (Sandimmun 50 mg/ml) at a dose of 10 mg/kg [32].

### Smear preparation of VM cell suspension and Immunocytochemistry

Smear preparation of the graft tissue prior to transplantation was prepared by fixing the cells with 4% paraformaldehyde (PFA) for 20 minutes at room temperature. The cells were washed twice with deionized water and resuspended to a final concentration of 2×10^4^ cells/µl. Two spots of 10 µl each of the cell suspension were added to gelatin-coated microscope slides, spread with the help of a pipette tip. They were then let to dry and stored at 2–8°C until processing for immunocytochemistry. Briefly, cell smears were washed three times with TBS before endogenous peroxidase was blocked with 3% of Triton ×100 in TBS for 10 min. Subsequently they were blocked for 1 h with 5% normal goat serum in PBS with 0.3% of Triton ×100 at room temperature. Primary and secondary antibodies were diluted in the serum blocking solution. Primary TH (mouse anti-TH, 1∶500, Sigma) antibody was applied overnight at 4°C. Secondary Alexa-555 conjugated goat anti-mouse antibody was applied at a dilution of 1∶2000 for 1 h 30 min at room temperature, 4′,6-diamidino-2-phenylindole (DAPI) was used for nuclear counterstaining.

### Tissue processing and Immunohistochemistry (IHC)

The animals were sacrificed 6 weeks after grafting procedure under deep thiopental (120–150 mg/kg) anaesthesia before perfusion using 50 ml of ice-cold 0.9% sodium chloride, followed by 200 ml ice-cold 4% PFA in phosphate buffered saline (PBS). The brains were removed, postfixed for 12 h in 4% PFA at 4°C and cryoprotected for 24–36 h in 30% sucrose. They were then frozen in isopentane (−40°C) on dry ice and kept at −80°C. Brains were cut on a cryotome, coronal sections were collected in 6 series at a thickness of 40 µm. Free-floating sections were washed three times with PBS before endogenous peroxidase was blocked with 3% H_2_O_2_ for 10 min. Washing was repeated after each step of the IHC protocol. Serum blocking was carried out with 5% normal goat serum and PBS with 0.3% of Triton ×100 at room temperature for 1 h. Primary and secondary antibodies were diluted in the serum blocking solution. Primary antibodies were applied overnight at 4°C.

One series of sections was processed for TH (mouse anti-TH, 1∶1000, Sigma), a second one for GFP (mouse anti-GFP, 1∶1000, Serotec) and a third one for GFAP (rabbit anti-GFAP, 1∶500, DAKO). For TH and GFP stainings, matching biotinylated secondary antibodies were applied at a dilution of 1∶200 for 1 h 30 min at room temperature. Followed by a 1 h incubation with streptavidin-horse-radish peroxidase complex (Vectastain Elite ABC Kit, Vector Laboratories) and subsequent exposure to 3,3′-diaminobenzidine (Fast DAB Tablets, Sigma) under microscope control. The sections were further analysed stereologically with Stereo Investigator Software on a Nikon E800 microscope.

Overnight incubation with primary GFAP antibody was followed by incubation with Alexa-555 conjugated goat anti-rabbit secondary antibody, at a dilution of 1∶2000 for 1 h 30 min at room temperature and 4′,6-diamidino-2-phenylindole (DAPI) was used for nuclear counterstaining.

### Cell counts and stereological analysis

The percentage of TH-positive cells in the VM cell suspension (pre–grafting) was determined in samples from three independent experiments. Therefore, images for quantification of GFP cells were randomly taken. The number of DAPI-positive nuclei was counted followed by counting TH-positive cells.

Quantification of TH and GFP cells in the transplant (post-grafting) was determined stereologically by optical fractionator. Percentage of cell death of TH and GFP was quantified according to the following formulas:
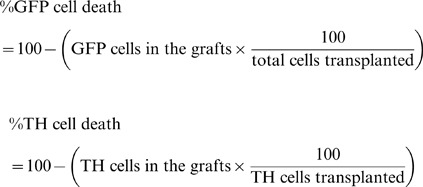


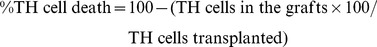
Quantification of GFAP positive cells in the graft was assessed from images randomly taken through the entire graft. In each image, the number of DAPI-positive perinuclear GFAP staining was first counted and then followed by counting GFP cells expressing GFAP. Pictures were taken with an ApoTome Imaging System based on Axio Observer Z1 (Zeiss) using AxioVision software.

### Graft and substantia nigra pars compacta (SNpc) dissection

On the 6^th^ week post-transplantation the animals were sacrificed by deep thiopental (120–150 mg/kg) anaesthesia. The animals were immediately decapitated and brains were removed and cut into 400 µm slices using a tissue chopper (McIlwain Tissue Chopper 10184-220). With the use of high magnification fluorescent stereomicroscope (Leica), grafts were identified and dissected manually using a scalpel.

After an injection of thiopental (120–150 mg/kg) anaesthesia, adult GFP mice were decapitated and brains were removed. Using a mice brain slice matrix, 500 µm slices were cut and with the help of anatomical coordinates (The Mouse Brain in Stereotaxic Coordinates, Second Edition, Paxinos and Franklin) the SNpc was localised and dissected.

### Quantitative Real-Time PCR

Samples of the VM cell suspension as well as dissected grafts and the SNpc tissue of adult GFP mice where immediately transferred into lysis buffer for RNA extraction and messenger RNA (mRNA) was isolated using Dynabeads® Oligo (dT) 25 (Invitrogen Corporation, Paisley, UK) following the manufacturer's protocol. The eluted mRNA was immediately used for cDNA synthesis according to High-Capacity cDNA Reverse Transcription Kit protocol (Applied Biosystems, Warrington, UK). The levels of cDNA were assessed by quantitative real-time PCR using Fast SYBR® Green Master Mix (Applied Biosystems, Life Technologies Corporation, Carlsbad, California, USA). Standard curves and melting curves were determined for each set of primers to confirm that a single amplicon was generated. The results were normalized to glyceraldehyde-3-phosphate dehydrogenase (Gapdh) or eGfp and expressed as ΔCt values (low ΔCt levels indicate high expression). Relative expression ratios were calculated by the ΔΔCt method [33]. Detailed information about the mouse primers is provided in Table 1.

**Table 1:** List of primers′ sequences used for qRT-PCR.

Gene Name	Forward Primer	Reverse Primer
Dat	5′-GGTCCTTCCGAGAGAAACTG-3′	5′-TCTCCTTCCACTTTACACCAAC-3′
eGfp	5′-AAGCAGAAGAACGGCATCAAG-3′	5′-GTGCTCAGGTAGTGGTTGTC-3′
En1	5′-GTCCGTCCTCTGGTCCAC-3′	5′-CGTGATATAGCGGTTTGCCTG-3′
Foxa2	5′-GTACTCCAGGCCTATTATGAAC-3′	5′-TTCCTCAAAGCTCTCCCAAAG-3′
Fgf2	5′-CTGGCTTCTAAGTGTGTTACAG-3′	5′-ATACTGCCCAGTTCGTTTCAG-3′
Fgf8	5′-CCTTCGCGAAGCTCATTGTG-3′	5′-AGTCCTTGCCTTTGCCGTTG-3′
Gad1	5′-ATGGTGAGCCTGAGCACAC-3′	CCACCATGGTTGTTCCTGAC
Gapdh	5′-AGGGCTCATGACCACAGTC-3′	5′-CAGCTCTGGGATGACCTTG-3′
Gfap	5′-CGGCACGAACGAGTCCCTA-3′	5′-CTAGCTTAACGTTGAGTAGATCC-3′
Lmx1a	5′-AGCGAGCCAAGATGAAGAAG-3′	5′-GAGTTGTAGACGCTCTGTTC-3′
Mash1	5′-GGAAGCAGGATGGCAGCAG-3′	5′-GAACATTGCATCTTAGTGAAGGTG-3′
MhcI	5′-ACATGGAGCTTGTGGAGACC-3′	5′-CCACATGGCATGTGTATTTCTG-3′
MhcII	5′-CAGTGTCCAGAGGTTTGCC-3′	5′-GTGGAAAGAGGTTGCTGATG-3′
Ngn2	5′-GCGTAGGATGTTCGTCAAATC-3′	5′-CAGCAGCATCAGTACCTCC-3′
Osp	5′-CCTTATTCTGCTGGCTCTCTG-3′	5′-CACCCACCAGGCACATCAC-3′
Pitx3	5′-CGTGCGGGTGTGGTTCAAG-3′	5′-CGGGTACACCTCCTCGTAG-3′
Sert	5′-GGTTCTATGGAATCACTCAGTTC-3′	5′-GATGAACAGGAGAAACAGAGG-3′
Shh	5′-GAAAGCAGAGAACTCCGTGG-3′	5′-AGGAAGGTGAGGAAGTCGC-3′
Th	5′-AAGGACAAGCTCAGGAACTATG-3′	5′-GCATTTAGCTAATGGCACTCAG-3′
vGlut2	5′-GACAAAGAATAAGTCCCGTGAAG-3′	5′-TCTCTCCTGAGGCAAATAGTG-3′
Vmat2	5′-GTATGCTATCGGTCCCTCTG-3′	5′-GAGTGTACATCTTTGTCTTAATGG-3′

### Statistics

All data are presented as mean ± SEM. The Statview (version 5.0.1) software was used (SAS Institute, Carry NC). The rotation differences between groups were analyzed using two-way analysis of variance (ANOVA) including the factors treatment and time with a post hoc Bonferroni for comparing grafted versus sham animals and pre versus post grafting time points. Two-tailed student's t-test or one–way analysis of variance (ANOVA) followed by a post hoc Student-Newman-Keuls test were used to compare gene expression between experimental groups. The level of statistical significance was set at p<0.05.

## Results

### Amphetamine-induced rotation behavior

**Figure 1:**
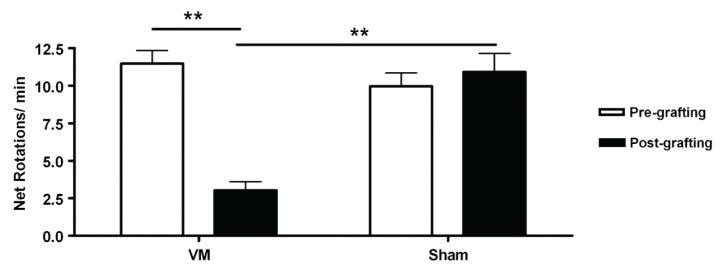
Functional impact of intrastriatal grafts. Amphetamine-Induced Rotation (2.5 mg/kg i.p injection) prior to grafting and 6 weeks post-transplantation is shown as net rotations/minute (over 90 min) for the graft group (n = 15) and the sham control group (n = 7). Only animals receiving intrastriatal VM grafts showed a recovery on motor behavior at the post-grafting time point (6 weeks). The data are presented as mean ± SEM. ** p<0.001 by Bonferroni post hoc test. SEM, standard error of the mean; VM, ventral mesencephalon.

In order to assess the functional effect of the VM grafts, amphetamine-induced-rotation was tested before and 6 weeks post-transplantation and compared to sham-grafted animals. Before grafting, sham (n = 7) and VM grafted animals (n = 20) displayed similar ipsilateral rotation behavior (p>0.05). Conversely, six weeks after transplantation (post-grafting) only VM grafted animals (n = 15) showed a significant reduction of rotation behavior (p<0.001) (Figure 1).

### Immunohistochemistry

**Figure 2:**
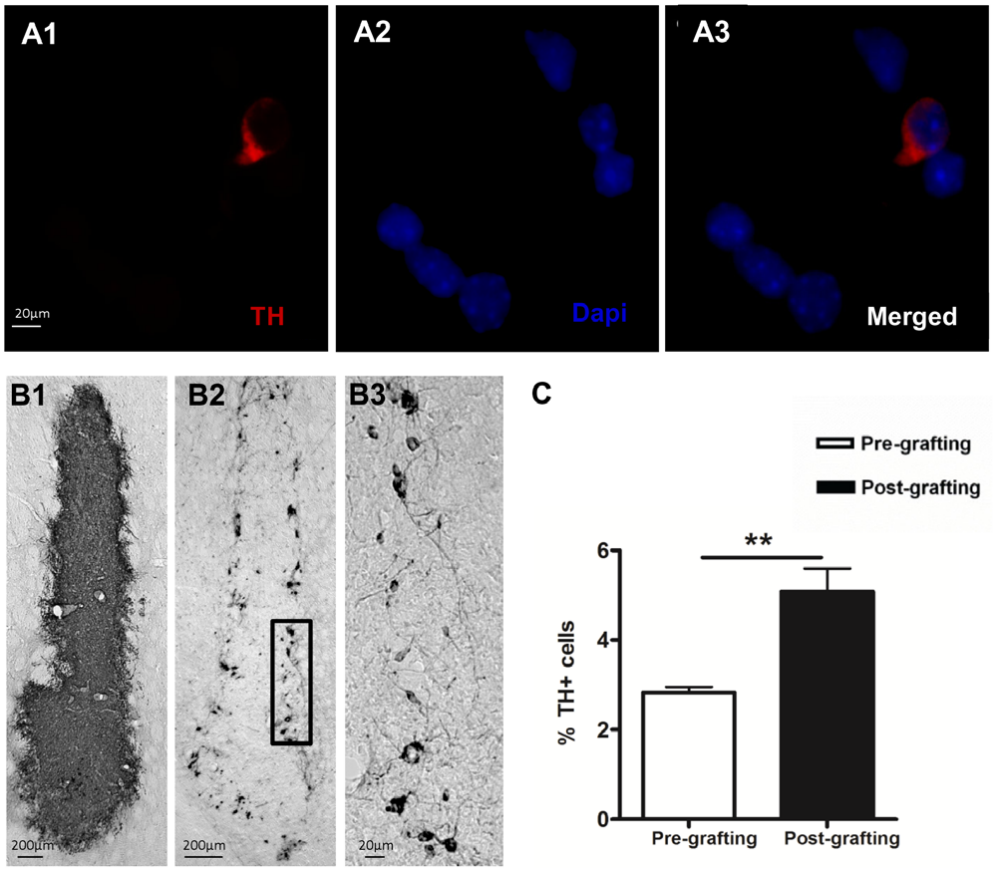
Increased TH-positive cells post-grafting. (A) Smear preparation of VM cell suspension. (**A**) TH staining (A1). DAPI staining (A2). Merged pictures (A3). GFP staining of grafts (**B1**), TH staining of grafts (**B2**). Higher magnification of the marked area showing TH cells extending axons which re-innervate the surrounding striatum (**B3**). (**C**) **Percentage of TH+ cells pre- and post-grafting.** Data are presented as mean ± SEM, n = 6. ** p<0.01 by two-tailed student's t-test. SEM, standard error of the mean.

The 6-OHDA lesion produced an almost complete loss of DAergic neurons in the ipsilateral SNpc (mean of 97%±3 compared to the contralateral side), resulting a depletion of striatal TH projections. Staining of the smear preparation of the VM cell suspension (Fig. 2 A) showed that 2.8±0.1% of the cells were TH positive. Therefore, from the 400 000 cells transplanted cells 11 300 were TH positive.

Six weeks post transplantation, animals had an average of 23610±6332 GFP cells within the graft (Figure 2 B1) indicating a death rate of 94±2%. Immunohistochemistry for TH (Fig. 2 B2 and B3) revealed that the grafts contained 1137±220 positive cells, indicating a death rate of 90±2% of DAergic cells. Two-tailed student's t-test revealed no significant difference of death rates of GFP versus TH cells (p>0.05). Post-grafting the TH positive cells represented 5.1±0.5% of the total GFP cells showing a near 2-fold increase of the ratio of TH/GFP cells in relation to the pre-grafting (2.8±0.1%) levels (p<0.01) (Figure 2 C).

### Molecular analysis of the grafts

Six weeks post-transplantation, grafts from half of the animals showing at least 40% reduction on the amphetamine-induced rotation behavior, were dissected (n = 7) and the tissue was analyzed by qRT-PCR. Due to the manual excision method, dissected grafts were contaminated with host tissue. Therefore, expression of all markers was first investigated in striatum of sham animals. When markers were detected in striatum of sham animals, expression values in the dissected grafts were normalized to Gapdh. In this case, the marker expression was only considered when expression values in the graft were significantly different from the striatum of sham animals. When the marker expression was not detected in the striatum of sham animals, expression values in grafted animals were normalized to Gfp.

#### The mDAergic phenotype

**Figure 3:**
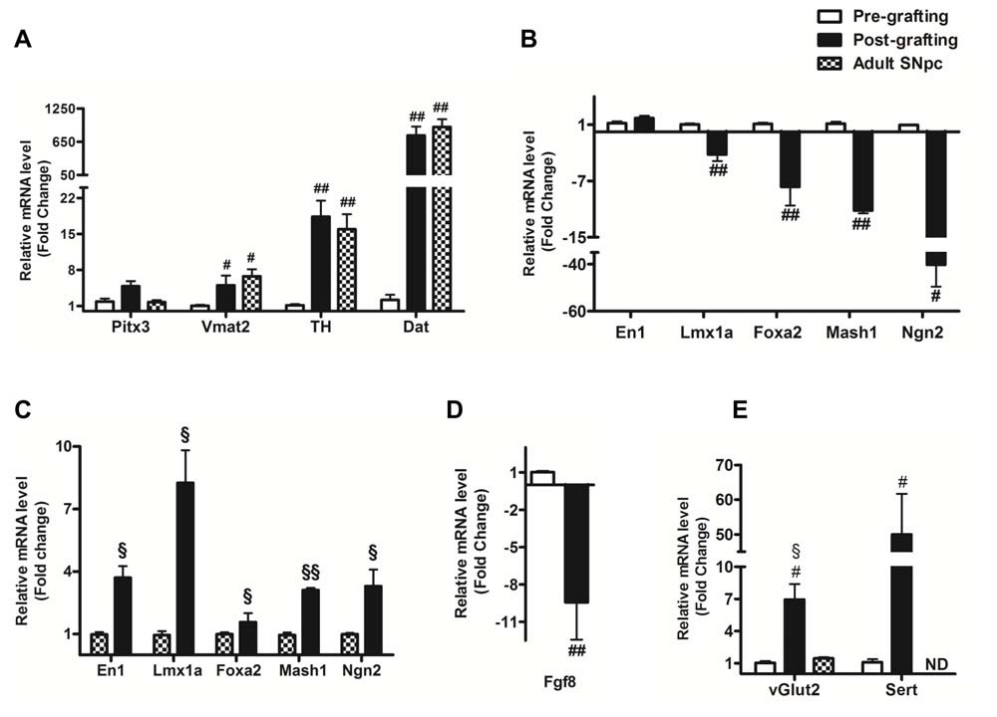
Molecular analysis of the neuronal populations pre-, post- grafting and in adult SNpc. (**A**) **Increased expression of mature DAergic markers post-grafting and in the SNpc.** Relative mRNA expression levels of the mature DAergic markers Pitx3, Vmat2, TH and Dat, 6 weeks post-grafting and in adult SNpc as compared to the mRNA levels pre-grafting. (**B**) **Decreased expression of DAergic progenitor markers post-grafting**. Relative mRNA expression levels of the DAergic progenitor markers En1, Lmx1a, Foxa2, Mash1 and Ngn2 post-grafting as compared to pre-grafting. (**C**) **Higher expression of DAergic progenitor markers in grafts (post-grafting) relatively to adult SNpc.** Relative mRNA expression levels of the DAergic progenitor markers En1, Lmx1a, Foxa2, Mash1 and Ngn2 post-grafting as compared to adult SNpc. (**D**) **Decreased expression of Fgf8 post-grafting.** Relative mRNA expression levels of Fgf8 post-grafting as compared to the mRNA levels pre-grafting. (**E**) **Increased expression of vGlut2 and Sert post-grafting.** Relative mRNA expression levels of vGlut2 and Sert post-grafting and in adult SNpc as compared to the levels pre-grafting. Levels of expression are presented as mean ± SEM and normalized to eGfp; n = 3 to 7; # p<0.05; ## p<0.01 significantly different from pre-grafting by Student-Newman-Keul's post hoc test for (A) and (E) or by student t-test for (B) and (D). § p<0.05; §§ p<0.01 significantly different from adult SNpc by Student-Newman-Keul's post hoc test for (E) and by student t-test for (C). SEM, standard error of the mean; ND, Not detected.

When compared with the VM cell suspension (pre-grafting), grafts surviving *in vivo* showed a significant increase in the expression of the markers for mature DAergic neurons: Vmat2, TH, and Dat (Figure 3 A). Additionally, we assessed the expression of the same markers in adult mouse SNpc and there was no significant difference of expression levels between adult SNpc and VM grafts (post-grafting) (Figure 3 A). We also detected reduced expression of markers of the DAergic progenitor pool (En1, Lmx1a, Foxa2, Mash1 and Ngn2) post-grafting, when compared with the VM suspensions (pre-grafting) (Figure 3 B). However, these transcription factors were expressed at higher levels in the grafts compared to the adult SNpc (Figure 3 C).

In addition, compared to VM cell suspension (pre-grafting), there was a significant decrease in Fgf8 expression within the grafts (Figure 3 D). We also observed a reduced expression of Shh in the grafts compared to VM cell suspensions. However, the level of expression was not significantly different from the baseline mRNA levels found in the striatum of sham animals (Figure S1).

#### Beyond the mDAergic phenotype

In addition to the mDAergic markers, we analyzed the mRNA expression of a set of neuronal and glial markers, including Gad1 for GABAergic, VGlut2 for glutamatergic and Sert for serotonergic neurons, as well as Gfap for astrocytes and Osp for oligodendrocytes. In comparison with pre-grafting levels, we found a significant increase of expression of vGlut2 and Sert post-grafting. Furthermore, we noticed that the levels of expression detected were significantly higher (p<0.05) than the expression detected in the adult SNpc (Figure 3 E). We also detected a higher expression of Gad1 post-grafting but the values were not significantly different from the baseline mRNA levels found in the striatum of sham animals (Figure S2).

**Figure 4:**
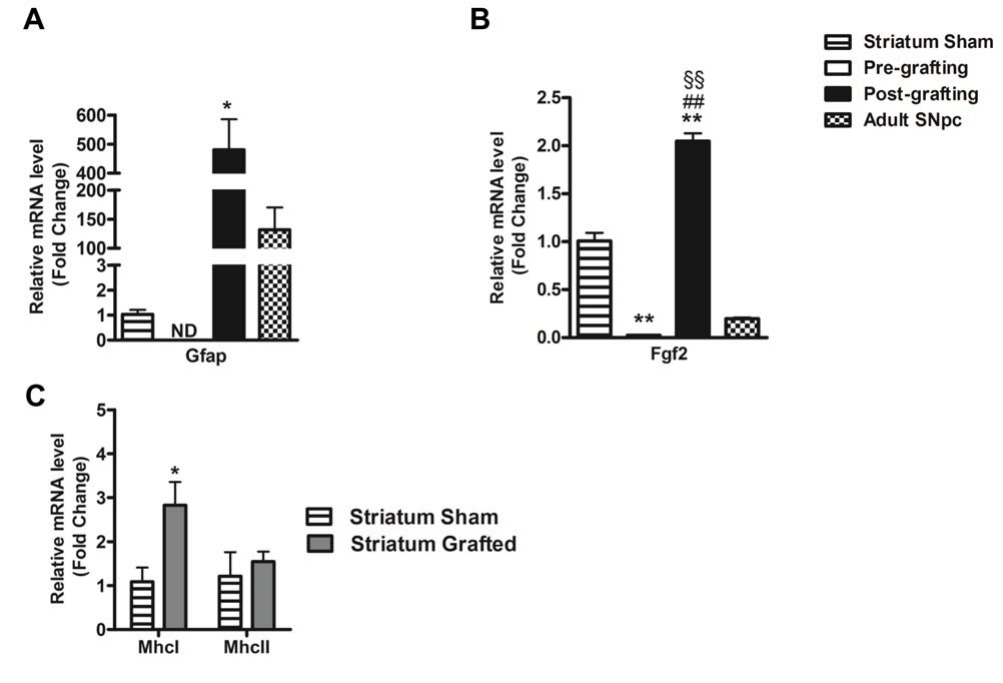
Molecular analysis of Gfap, Fgf2, MhcI and MhcII pre-, post- grafting and in adult SNpc. (**A**) **and** (**B**) **Increased expression of Gfap and Fgf2 post-grafting**. Relative mRNA expression levels of Gfap and Fgf2 pre-, post-transplantation and in adult SNpc as compared to sham animals. (**C**) **Increased expression of Mhc I in striatum of grafted animals.** Relative mRNA expression levels of MhcI and MhcII in the striatum surrounding the fluorescent transplant of the grafted animals (Striatum Grafted) as compared to the striatum of sham animals (Striatum Sham). Levels of expression are presented as mean ± SEM and normalized to Gapdh; n = 3 to 7; * p<0.05, ** p<0.01 significantly different from sham by Student-Newman-Keul's post hoc test (A and B) or student t-test (C), ## p<0.01 significantly different from pre-grafting and §§ p<0.01 significantly different from adult SNpc by Student-Newman-Keul's post hoc test. SEM, standard error of the mean. ND, not detected.

**Figure 5:**
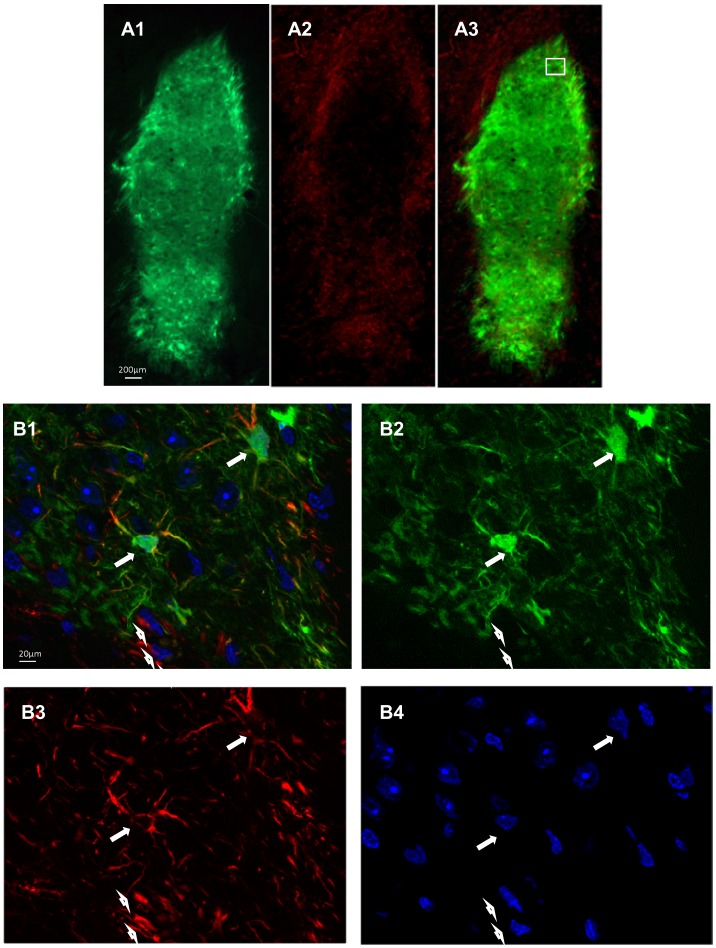
Presence of donor derived Gfap-positive cells within the grafts. (**A1**) **GFP expressing grafts** GFAP staining (**A2**). Merged pictures (**A3**). Higher magnification of the marked area showing donor derived (GFP) GFAP-positive cells within the grafts (long arrows). Host derived (non GFP) GFAP-positive cells are also shown (short arrows). (**B1**). GFP expressing cells (**B2**) GFAP staining (**B3**). DAPI staining (**B4**).

We found no difference in the expression of Osp between VM cell suspension (pre-grafting), sham striatum, grafts and adult SNpc (Figure S3). A high level of Gfap expression was detected post-grafting contrasting the lack of expression of this marker in the VM cell suspensions tissue (pre-grafting) (Figure 4 A). Gfap mRNA was detected in the striatum of sham animals, however, the level of GFAP expression detected post-grafting was significantly elevated. Higher expression of Gfap was also detected in the grafts when compared with the adult SNpc, but this difference was not significant (Figure 4 A). GFAP protein expression (Figure 5 A) showed the presence of donor derived GFP and GFAP double positive cells within the grafts (Figure 5 B) which accounted for 63±7% of total GFAP-expressing cells.

Basic fibroblast growth factor (FGF2) is a growth factor involved in the development, maintenance, and survival of the nervous system. We found that Fgf2 mRNA expression was significantly increased post-grafting (p<0.05) relatively to the pre-grafting levels and grafts exhibit a higher expression of Fgf2 when compared to the adult SNpc (Figure 4 B).

Finally, there was a significantly higher expression of the major histocompatibility complex class I (Mhc I) in the striatum of grafted animals relative to sham grafted animals. No difference was found in Mhc II (Figure 4 C).

## Discussion

Translational research on cell therapy in PD has been hindered by the highly variable outcome of the clinical trials. Owing to the different tissue preparations between studies, it is likely that the large variation in the outcome between patients is in part associated with the lack of standardization and quality control requirements for VM grafts [34], [35]. In order to advance our understanding of the molecular and cellular changes occurring in embryonic VM grafts *in vivo*, we analyzed their molecular signature prior to and six weeks post-grafting in the unilateral 6-OHDA rat model. We also performed a comparative and quantitative analysis of the molecular “fingerprint” between grafts and DAergic neurons of an adult SNpc. Immunocytochemical analysis of embryonic VM cell suspensions before implantation versus grafts immunohistochemical analysis revealed a 2-fold increase of the TH positive cells, indicative of post-implantation DAergic differentiation. Tissue analysis by qRT-PCR confirmed these results, showing that, when compared with the developing VM, implanted grafts had a significant increase of Vmat2, TH, and Dat expression associated with a decrease in the expression of DAergic progenitor markers. Implanted grafts exhibited a mature molecular DAergic phenotype similar to the adult SNpc. However, the level of expression of the progenitor markers was higher in the grafts than in the adult SNpc, suggesting that the differentiation process is incomplete 6 weeks post-implantation. We also detected an increase of expression of markers inherent to other neuronal populations post-grafting indicating that host brain is favorable to the development of these other lineages.

The optimal developmental age for harvesting ventral mesencephalic cells for transplantation in PD models is based on two principles. First, the cells need to be harvested before the TH neurons extend their axon to the striatum, otherwise the viability of the cells is jeopardized due to the physical trauma during the tissue preparation and surgical intervention [36]. Second, immature mesencephalic progenitors do not differentiate properly *in vivo* because they are not exposed to the necessary molecular cues in the host brain [15]. Therefore, transplantation studies in animal models of PD have recommended an optimum donor age between E14–E15 for rat [37] and E12–13 for mice [38] corresponding to the period close to the peak of neurogenesis [39]. We found that at E12.5, the VM tissue expressed a set of markers consistent with the presence of a heterogeneous population of cells at different stages of development and differentiation. In particular we detected expression of a combination of markers for DAergic proliferating progenitors including En1, Lmx1a, Foxa2, Mash1 and Ngn2. At the same time we found Pitx3, a marker for postmitotic precursors, as well as markers for fully differentiated DAergic neurons, such as TH, Vmat2, and Dat.

DAergic grafts induced an almost complete restoration of amphetamine-induced-rotation asymmetry and contained an average of 1137±220 of TH cells. By measuring the number of TH cells in VM cell suspension and the number of surviving TH neurons in the transplants we showed a 2 fold increase of DAergic cells post-implantation indicative of differentiation post-transplantation. qRT-PCR analysis corroborated these immunohistochemical data by showing that mRNA expression levels of TH, Dat and Vmat2 were significantly increased post-transplantation. The concomitant decrease of markers associated with the progenitor's pool such as En1, Lmx1a, Foxa2, Mash1, and Ngn2, suggests that progenitor cells differentiate in the graft to form TH expressing neurons. Although the pattern of expression of the fully differentiated DAergic neuronal markers (TH, Dat and Vmat2) was similar in grafts and adult SNpc, the DAergic progenitor markers, En1, Lmx1a, Foxa2, Mash1 and Ngn2 were higher in the grafts compare to naïve adult tissue. We propose that although some of the progenitors appear to have the potential to differentiate to TH neurons in the host brain; other progenitors fail to develop further. These progenitor cells may not be sufficiently committed at the time point of transplantation to finalize their differentiation processes *in vivo*. Alternatively, cell extrinsic cues for DAergic neuron differentiation in host striatum might be the limited factor. At E12.5 we still detected a considerable expression of endogenous Fgf8 in VM cell suspensions while Fgf8 was not expressed in sham animals and was significantly decreased post-grafting compare to pre-grafting.

In addition to the DAergic population, the developing VM transplants are composed of other neuronal population, including GABAergic and serotonergic neurons. It has been shown that these non-DAergic populations can make connections with the host striatum after grafting [40]. In our experiments we observed an increased expression of markers for 5-HT and glutamatergic neurons post-grafting when compared to pre-grafting levels confirming previous results that these non-DAergic populations can develop in grafts [40]. In the clinical transplantation trials, protocols regarding tissue dissection and handling, fetal donor age and tissue storage differed among centers [35], resulting in substantial variability to the composition of the grafts. Co-grafted 5-HT neurons have been implicated in the development of GIDs [41]. In the light of our results, future studies should also investigate the functional implications of co-grafted glutamatergic neurons in VM grafts. We did not detect expression of mature markers for these populations in the VM cell suspensions. Therefore markers for immature cells from these lineages should be included in future experiments.

Gfap mRNA as a marker for mature astrocytic glial cells was undetectable in the VM cell preparations pre-grafting. However, post-grafting, a high GFAP mRNA expression was determined and the levels were significantly higher than in tissue from sham grafted animals. This differential Gfap expression between post-grafting and sham animals might in part be explained by stronger gliotic reaction to the grafted tissue compared to sham operated animals. However, immunohistochemistry for GFAP confirmed the presence of a large number of GFP positive, hence donor derived, GFAP-positive cells within the grafts. Since Gfap was not detected pre-grafting, we conclude that gliogenesis occurs in the graft post implantation.

Astrocytes within and surrounding the graft may have detrimental effects on grafted neurons and precursors by releasing pro-inflammatory molecules [42]. However, astrocytes might also contribute to neuroprotective mechanisms by detoxifying oxygen free radicals or secreting neurotrophic factors such as glial-cell-line-derived neurotrophic factor [43]. Pre-treatment of embryonic VM grafts or intracerebral infusion of Fgf2, a growth factor also secreted by astrocytes [44], promotes the survival of the transplanted cells [45]. In addition, intrastriatal infusions of Fgf2 induced recovery of DAergic fibers and caused an increase of the dopamine levels in the striatum of mice treated with MPTP [46]. We show an increased expression of Fgf2 post-grafting relative to pre-grafting levels. It appears likely that this increase is correlated with increased gliogenensis.

The ambiguous results of the clinical trials so far provide strong indication that cell preparations for transplantation therapy in PD need to be optimized and standardized before a clinically competitive cell therapy can be achieved [47]. Our study contributes to this aim by showing how qRT-PCR can be used to characterize gene changes in intracerebral VM grafts. This simple methodology requiring only a very limited number of graft cells appears useful to control the composition of the VM suspensions before grafting and to standardize the protocols for cell transplantation in PD. The same technique is useful to characterize cell fate decisions after grafting. Our results show that, besides the DAergic phenotype, embryonic VM grafts are composed of other neural population at the time of transplantation and that the host brain enables the development of several lineages including glutamatergic, serotonergic and glial cells. These populations are most likely contributing to the variable functional outcome of transplantation studies and to side effects. Finally, we believe that it is crucial to evaluate expression levels of molecules in host brain that affect the survival, proliferation and differentiation of the grafts. Again a quantitative analysis of components of the transcriptome of the host striatum with or without the grafted cells as exemplified in our study could be the method of choice.

## Supporting Information

Figure S1 Relative mRNA expression levels of Shh pre-, post-transplantation and in adult SNpc as compared to sham animals. Levels of expression are presented as mean ± SEM and normalized to Gapdh; n = 3 to 7. ** p<0.01 significantly different from sham, ## p<0.01 significantly different from pre-grafting and §§ p<0.01 significantly different from adult SNpc by Student-Newman-Keul's post hoc test. SEM, standard error of the mean.
(TIF)

Figure S2 **Relative mRNA expression levels of Gad1 pre-, post-transplantation and in adult SNpc as compared to sham animals.** Levels of expression are presented as mean ± SEM and normalized to Gapdh; n = 3 to 7. * p<0.05 significantly different from sham and # p<0.05 significantly different from pre-grafting by Student-Newman-Keul's post hoc test. SEM, standard error of the mean.
(TIF)

Figure S3 **Relative mRNA expression levels of Osp pre-, post-transplantation and in adult SNpc as compared to sham animals.** Levels of expression are presented as mean ± SEM and normalized to Gapdh; n = 3 to 7. SEM, standard error of the mean.
(TIF)
