# Supplementary figures and images for: Cell Fate Analysis of Embryonic Ventral Mesencephalic Grafts in the 6-OHDA Model of Parkinson's Disease
Source: PLoS One. 2012 Nov 29;7(11):e50178. doi: 10.1371/journal.pone.0050178 (PMC3510255; doi:10.1371/journal.pone.0050178)

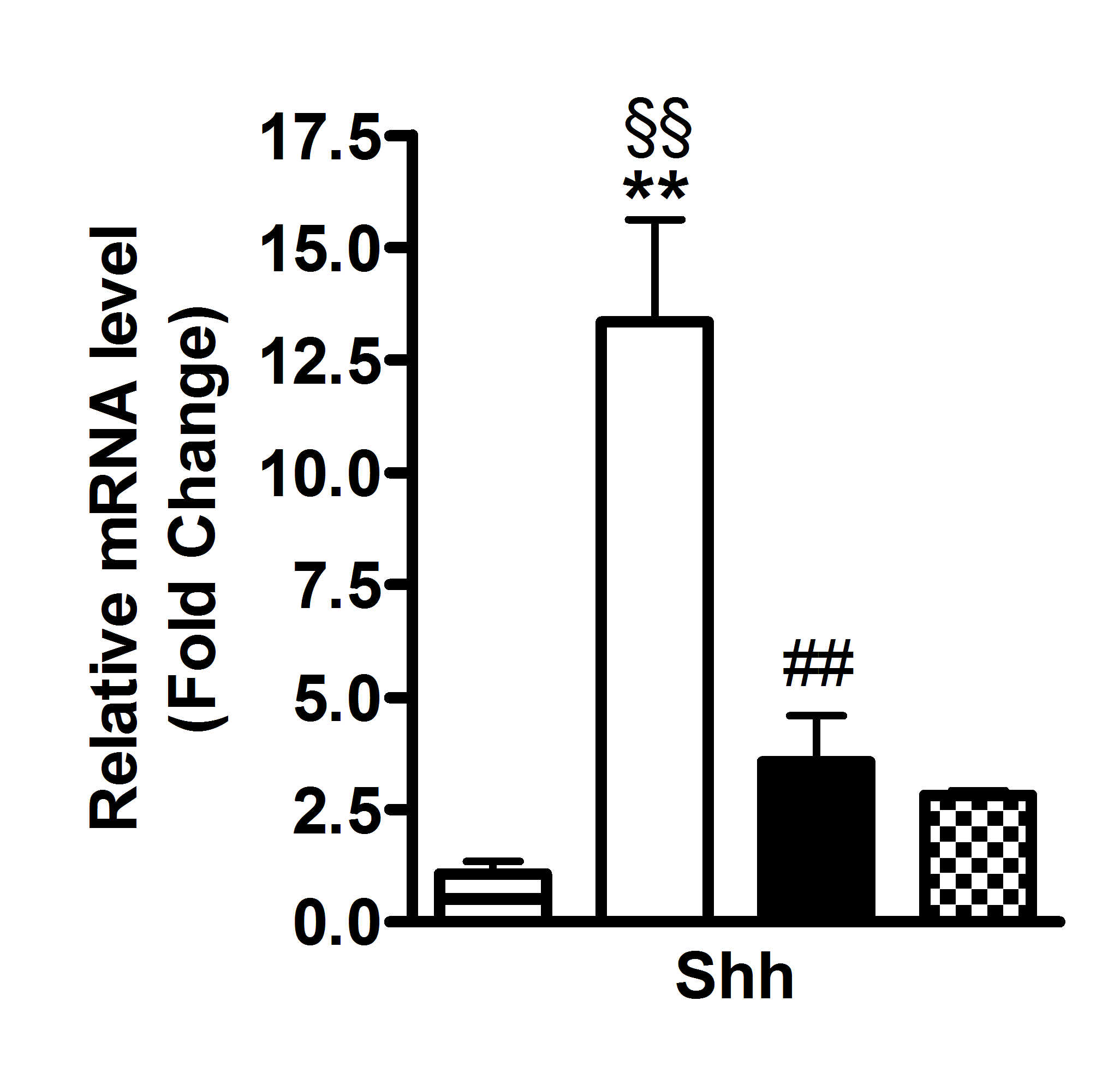

Supplement: Figure S1 — Relative mRNA expression levels of Shh pre-, post-transplantation and in adult SNpc as compared to sham animals. Levels of expression are presented as mean ± SEM and normalized to Gapdh; n = 3 to 7. ** p<0.01 significantly different from sham, ## p<0.01 significantly different from pre-grafting and §§ p<0.01 significantly different from adult SNpc by Student-Newman-Keul's post hoc test. SEM, standard error of the mean. (TIF) [file pone.0050178.s001.tif]

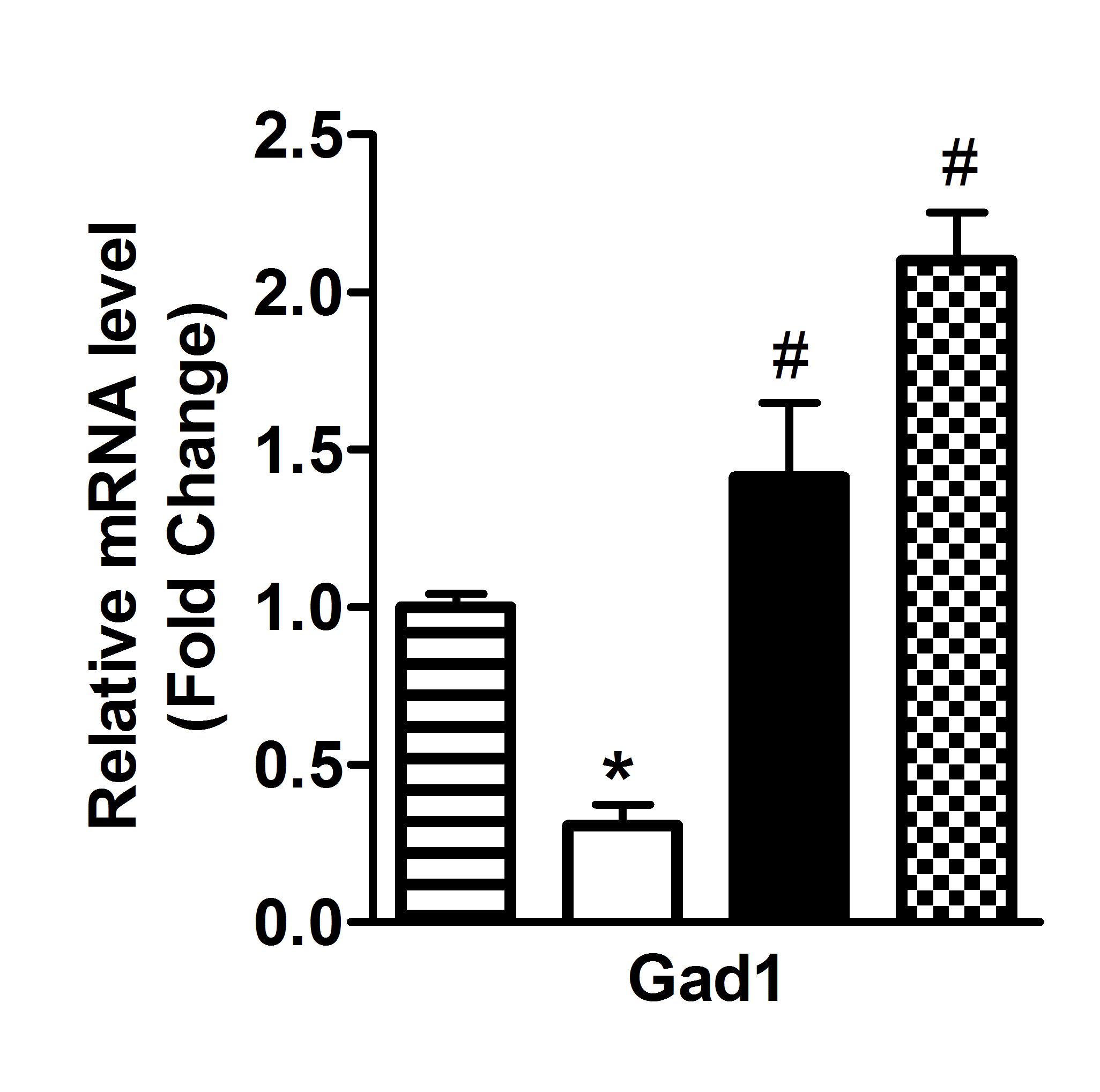

Supplement: Figure S2 — Relative mRNA expression levels of Gad1 pre-, post-transplantation and in adult SNpc as compared to sham animals. Levels of expression are presented as mean ± SEM and normalized to Gapdh; n = 3 to 7. * p<0.05 significantly different from sham and # p<0.05 significantly different from pre-grafting by Student-Newman-Keul's post hoc test. SEM, standard error of the mean. (TIF) [file pone.0050178.s002.tif]

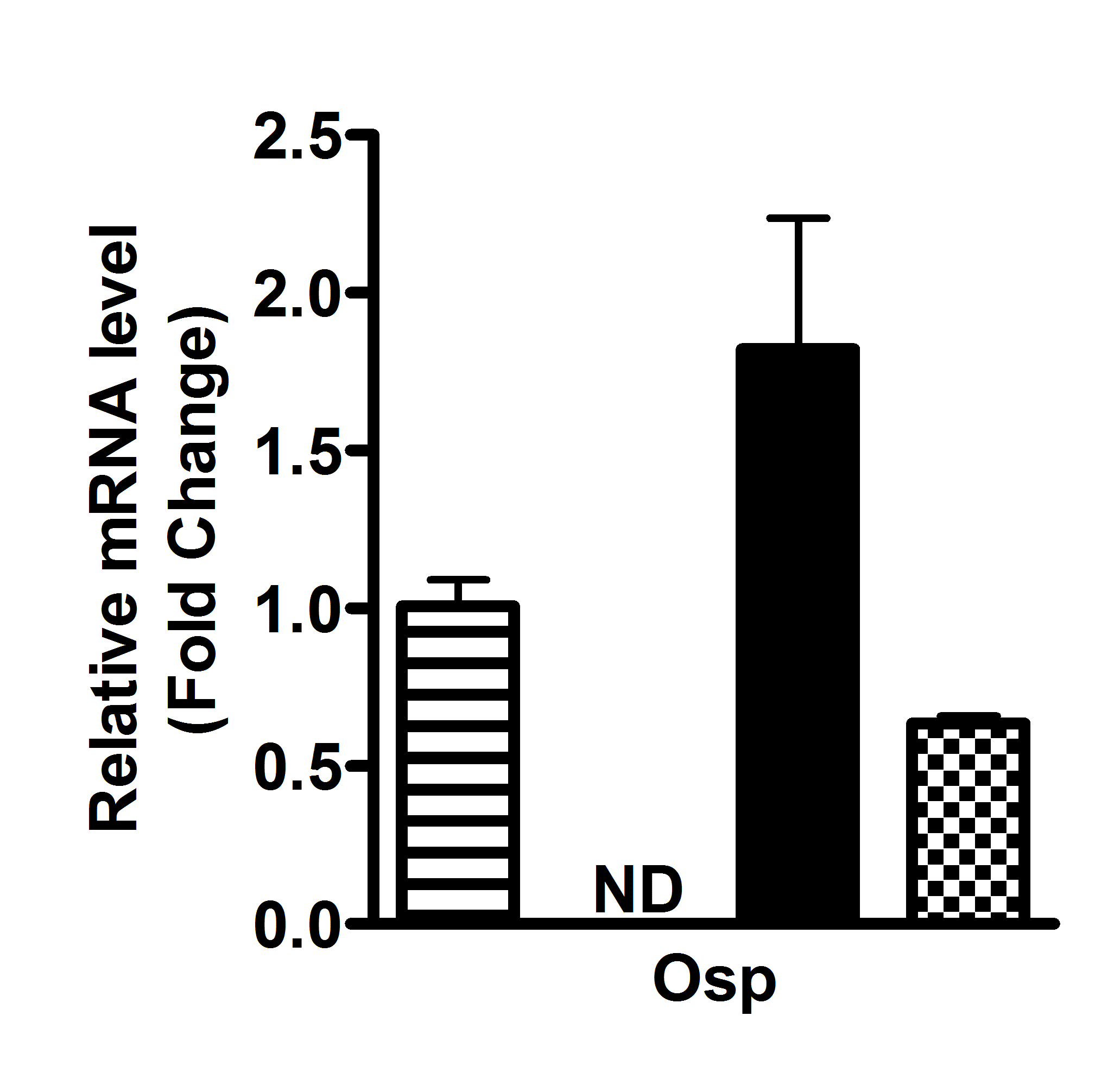

Supplement: Figure S3 — Relative mRNA expression levels of Osp pre-, post-transplantation and in adult SNpc as compared to sham animals. Levels of expression are presented as mean ± SEM and normalized to Gapdh; n = 3 to 7. SEM, standard error of the mean. (TIF) [file pone.0050178.s003.tif]
